# A meta-analysis of the relationship between bullying and non-suicidal self-injury among children and adolescents

**DOI:** 10.1038/s41598-022-22122-2

**Published:** 2022-10-14

**Authors:** Haitao Huang, Yueming Ding, Xiao Wan, Yipei Liang, Yiming Zhang, Guangli Lu, Chaoran Chen

**Affiliations:** 1 Institute of Nursing and Health, School of Nursing and Health, Henan University, Kaifeng, 475004 People’s Republic of China; 2 Institute of Business Administration, School of Business, Henan University, Kaifeng, 475004 People’s Republic of China

**Keywords:** Human behaviour, Psychology

## Abstract

Non-suicidal self-injury (NSSI) has attracted increasing attention due to its high detection rate, high risk and high repeatability. There is a need for the early identification of preventable occurrence factors, which is necessary to facilitate screening and intervention, especially to facilitate the early detection of high-risk individuals. This research aims to investigate the relationship between bullying behaviour and non-suicidal self-injury among children and adolescents by means of meta-analysis. The PubMed, Embase, Web of Science, SCOPUS, PsycINFO, CKNI and WAN FANG databases were searched from inception to 14 December 2021 for studies that explored the relationship between bullying behaviour and NSSI among children and adolescents. A total of 29 articles met the inclusion criteria of the meta-analysis, and 54 independent effect sizes were obtained, including 53,501 subjects. Victims [OR 2.46 (95% CI 2.14–2.83); *p* < 0.001], bullies [OR 2.12 (95% CI 1.37–3.27); *p* < 0.001], and bully-victims [OR: 2.98 (95% CI 1.85–4.82); *p* < 0.001] were more likely to have NSSI than uninvolved children or adolescents. In addition, analyses showed the absence of publication bias. In the victim group, the older the age was, the lower the risk of NSSI (z = − 3.74, *p* = 0.00). Gender does not play a moderating effect on the association between bullying behaviour and non-suicidal self-injury. The relationship between involvement in bullying and NSSI was demonstrated. By taking measures to prevent bullying, the incidence of NSSI in children and adolescents can be potentially reduced.

## Introduction

NSSI involves to intentional direct self-harm to one's own body without suicidal thoughts, may include scratches, bumps, cuts, burns and other forms, and jt is not socially and culturally recognized^1^. The most recent version of the Diagnostic and Statistical Manual of Mental Disorders (DSM-5) introduces non-suicidal self-injury as a disorder for further exploration^2^. In a meta-analysis, the incidence of non-suicidal self-injury in adolescents was as high as 17.2%^3^. Liu et al.^4^ surveyed 2716 Chinese adolescents and found that 26.9% of the subjects had NSSI in the past year suggesting that paying attention to NSSI should be one of the important tasks for maintaining adolescent mental health. Although most people who repeat non-suicidal self-injury stop this behaviour within a few years, they usually follow a more chronic process, with approximately 20% of them continuing for more than 5 years^5^. NSSI is often strongly associated with depression or bipolar disorder in adolescents^6,7^. The reason may be that brain changes in early-onset individuals (especially adolescents) with mood disorders have a significant impact on negative experiences^8^. Studies have shown that adolescents with bipolar depression have more abnormalities in their brains than those with unipolar depression^8^. In addition, many empirical studies have indicated that NSSI will not only increases the risk of mental illness, but also significantly increases the risk of suicidal behavior^9,10^. The incidence of suicidal behaviour is frequently underreported. For example, studies have confirmed that more than 2% of traffic accidents are considered suicide behaviours^11^. However, car accident suicides are often reported as accidental events in national statistics, so this phenomenon may be underestimated^11^. Therefore, given the negative outcomes of NSSI, timely detection of risk factors for non-suicidal self-injury may be helpful for the formulation of prevention programs.

Within this context, several studies have shown that bullying behaviour is an important predictor of NSSI among children and adolescents^12–14^ There are cultural differences in the definition of bullying, however, bullying generally refers to repeated physical or verbal harassment and involves the imbalance of power between the bully and the bullied^15,16^. Participants in bullying are generally divided into three types: bully (bullying others), victims (suffering from bullying), and bullying-victims (both bullying and being bullied by others)^17^. Although the effects of participating in bullying are about the same, the incidence of bullying may vary according to the role played by individuals in bullying. According to related research, approximately 15–50% of children participate in bullying behaviour as a bully, victim or bullying-victim^18^. Adolescents who are bullied may use self-harm as a form of calling for help^16^. Self-harm may be a form of relief from bullying-related stress and is a kind of self-punishment of the individuals^19^. In addition, due to a lack of self-regulation and impulsiveness, bullies may also develop self-injurious behaviours^20^. Bully-victims are also highly likely to have risky behaviours, as they have the risk factors for both bullies and be bullied^21^.

Meta-analysis is a scientific research method that can summarizes the results of several studies to evaluate the overall effects. In this study, the aim was investigate the association between victims, bullies, bully-victims and NSSI in adolescents by conducting 3 meta-analyses. As publications are more likely to publish studies with positive results and reject papers without positive results, this may lead to potential bias. Therefore, publication bias of the results were also verified through several methods^22^. In addition, the moderating effects of sampling strategy, gender and age were tested by subgroup analysis or meta-regression analysis to determine whether the relationship between variables was moderated by the above factors.

## Methods

This study was conducted strictly according to the Preferred Reporting Items for Systematic Reviews and Meta-Analyses (PRISMA) lists. See Supplementary file 1 for specific PRISMA Checklist.

### Study retrieval and selection

**Figure 1:**
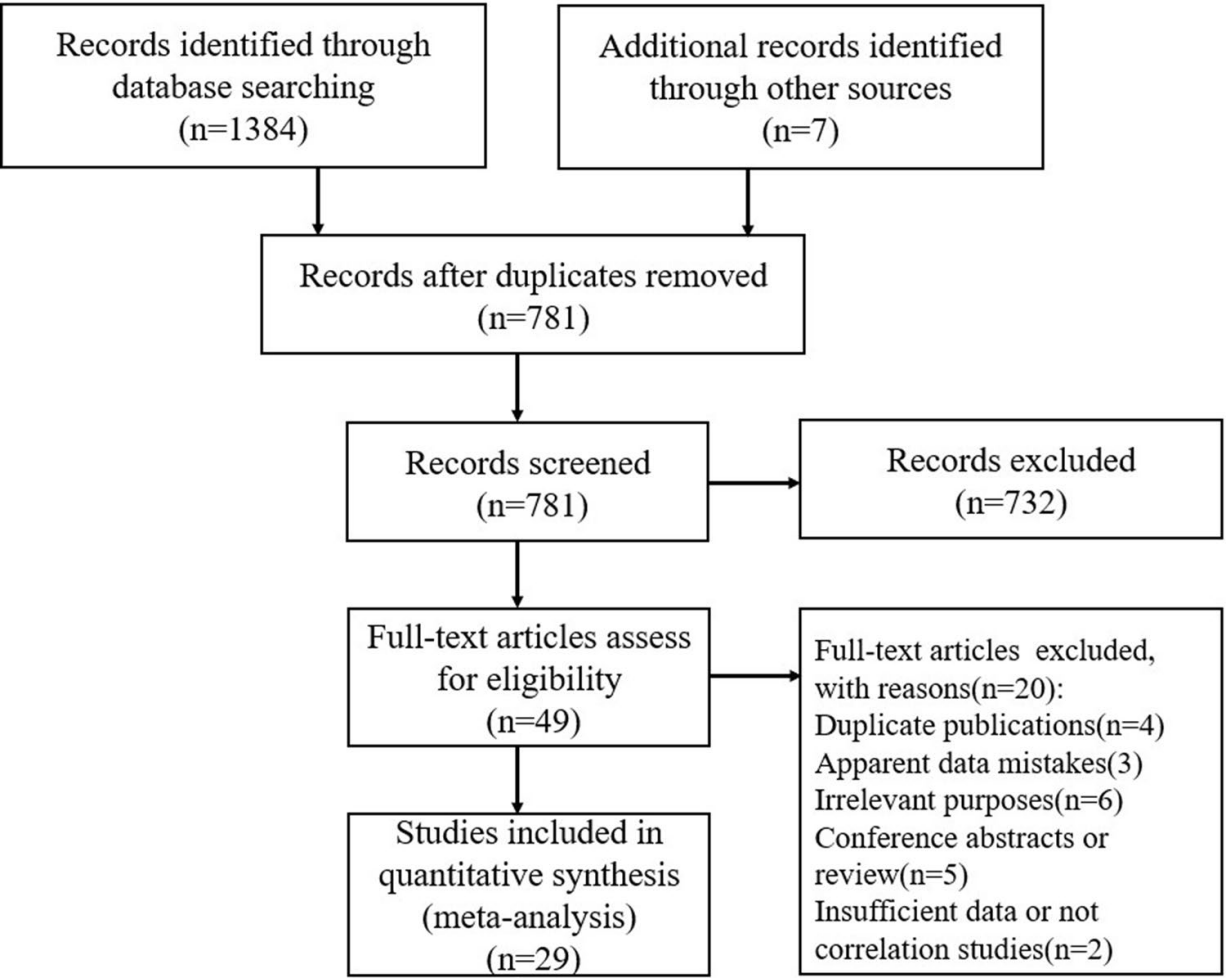
Flow diagram of the search results.

The PubMed, Embase, SCOPUS, Web of Science, PsycINFO, CKNI and WANG FANG databases were searched. These databases have the characteristics of being widely used, extensive and authoritative. The search words were ("bully*" OR" bullie*" OR "peer victim*" OR "peer harassment" OR "school violence" OR "mobbing "OR" ragging") and “self-harm*" OR "self-injur*" OR "self-cut*" OR "self-destruct*" OR "parasuicid*" OR "automutilat*" OR "auto-destruct*" OR "non-suicidal" OR "self-mutilation" OR "NSSI") (14 December 2021). See Supplementary file 2 for specific retrieval strategies. References to retrieved studies were also scanned to further identify studies that met the inclusion criteria. This search strategy yielded 1391 studies. Two researchers independently reviewed all references to find as many studies as possible that met the inclusion criteria. After the duplicate studies were removed with Endnote, 781 studies remained. The specific literature screening process is shown in Fig. 1. Only peer-reviewed studies published in journals were considered for inclusion. Only studies that provide an effect size for the relationship between bullying and NSSI were considered for inclusion; Therefore, studies that met the inclusion criteria either compared the NSSI of adolescents who participated in bullying to the NSSI of adolescents who did not participate, or treated bullying behaviour and NSSI as continuous variables, providing the degree of correlation between the two variables. Only studies that provide an effect size of the current link between bullying behaviour and NSSI were considered for inclusion. If only prospective or retrospective data were presented, the study was not considered for the time being. In previous meta-analyses, retrospective studies and prospective studies are usually tested separately from cross-sectional studies because the first two pay attention to how bullying affects victims' later lives, which is different from how bullying is directly related to NSSI. However, two studies^23,24^ provided both prospective and current linking data, so we chose to include the Time 1 section containing the current links between bullying behaviour and NSSI in the meta-analysis. Studies with participants over the age of 21 were excluded. In addition, only literature that clearly focuses on bullying was considered. The forms of bullying mainly included physical bullying (e.g., hitting, kicking), verbal bullying (e.g., name-calling, insulting), and psychological or social bullying (e.g., lying, spreading rumors). Studies focusing on victimization or aggression against non-peers were excluded. Research was only included if it focuses on actual NSSI behavior rather than thoughts or ideation of self-injury. Papers were coded as NSSI only when the interviewees of the study were explicitly required to report self-injury with no intention to die.

Only studies with a sample of children and adolescents from the community were considered for inclusion in the meta-analysis; papers focusing on clinical samples were excluded as conclusions drawn from clinical samples are not suitable for generalization in the general population. To prevent each researcher from being repeatedly included in our study, the articles using more respondents for the studies using the same dataset were included. Studies written in English and Chinese were eligible for inclusion. Two authors independently evaluated the literature according to the inclusion and exclusion criteria. Finally, 29 studies met our inclusion criteria. Table 1 shows the basic characteristics of the included literature.

**Table 1:** Studies included in the meta-analysis.

Authors	Country	N/%Female	Age	Sampling	Bully	NSSI
Hay et al.^25^	USA	426 (50%)	15 ± 2.18	Convenience sampling	Self-report	Self-report
					Six item scale including verbal physical and relational victimization	Respondents are asked how often they hurt themselves without wanting to die
Jutengren et al.^24^	Sweden	880 (49.32%)	13.72 ± 0.78	Convenience sampling of seven urban junior high schools	Self-report	Self-report
					Five items covering derogation, and three items covering relational, physical and verbal bullying	Used a revised version of the deliberate self-harm inventory
Noble et al.^26^	USA	1276 (72.6%)	Middle school: 13.80 ± 0.61	Randomized sample from each school and each grade	Self-report	Self-report
			High school; 16.25 ± 1.04		During the past 12 months, has someone bullied you on school property?	Participants are asked if they ever hurt themselves, with a follow-up question of whether this was with the intent to die
Bakken et al.^27^	USA	2548 (50%)	9th–12th grade	Random sampling of schools and classroom	Self-report	Self-report
					3 questions on general bullying, physical bullying and theft, all on school property	During the past 12 months, did you do something to purposely hurt yourself without wanting to die, such as cutting, scraping, or burning yourself on purpose?
Giletta et al.^28^	Netherlands, Italy, and USA	1862 (49%)	15.69 ± 0.87	Convenience sampling of 10 schools across three countries	Self-report	Self-report
					Three items from the revised Olweus Bully/Victim Questionnaire	Six item measure of self-injuring behaviours, without the intent to die
Claes et al.^13^	Belgium and Netherlands	785 (44.5%)	15.56 ± 1.32	Convenience sampling	Self-report	Self-report
					Bully/victim self-report questionnaire	Use of a self-harm inventory subscale: specified that self-injuring behaviors should be without the intent to die
Jantzer et al.^29^	Germany	647 (50.7%)	12.8 ± 1.95	Convenience sampling in the city of Heidelberg	Self-report	Self-report
					Revised bully/victim questionnaire	Frequency of self-harming behaviours without the intent to die
Garisch et al.^23^	New Zealand	1162 (43%)	16.35 ± 0.62	Convenience sampling	Self-report assessed using questions from Section D of the Peer Relations Questionnaire	Self-report assessed using the Deliberate Self-Harm Inventory– Short form (DSHI-s;)
Hamada et al.^12^	Japan	1840 (51.41%)	13.9 ± 0.2	Convenience sampling	Self-report	Self-report
					Bully/victim self-report questionnaire	Frequency of self-harming behaviours without the intent to die
Jiang et al.^30^	China	525 (43.04%)	12.97 ± 1.02	All 7th and 9th grade children in Foshan	Self-report	Self-report
					Participants were asked:“ How often has someone bully you"?	Participants reported the frequency with which they intentionally injured themselves without the intention to die, during the past year
Xavier et al.^31^	Portugal	854 (52.8%)	14.89 ± 1.79	Convenience sampling	Self-report	Self-report
					Peer Relations Questionnaire (PRQ)	Frequency of self-harming behaviours without the intent to die
Wright^14^	USA	96 (All boys)	14.03 ± 0.51	Convenience sampling	Self-report	Self-report
					two subscales included for this questionnaire, which assessed adolescents’ perpetration and victimization by bullying	Participants completed the Self-Harm Inventory, which consists of 22 yes/no items asking them if they ever intentionally engaged in the described behaviours, such ascutting. All behaviors were described as occurring without suicidal intent
Ji et al.^32^	China	679 (47%)	14.2	Convenience sampling	Self-report	Self-report
					Revised bully/victim questionnaire	Frequency of self-harming behaviours without the intent to die
Baiden et al.^33^	Canada	1650 (45.8%)	14.56 ± 1.79	Convenience sampling	Self-reporttwo items that asked for history of self-injurious behavior and the intent behind the self-injurious behavior. Those who engaged in self-injurious behavior with the intent to kill themselves were excluded	Self-report bullying victimization was measured in reference to lifetime as opposed to past year or past month
Thomas et al.^34^	Canada	2967 (48.4%)	14.6	random sample of house- holds with children and adolescents aged 4–17 years residing	Self-report	Self-report
					revised Olweus Bully/Victim Questionnaire	Participants were asked,‘Have you deliberately harmed or injured yourself without intending to end your own life during the past 12 months?
Jiang et al.^35^	China	1810 (44.5%)	Senior one and Senior two	Cluster sampling	Self-report	Self-report
					Revised bully/victim questionnaire	Have you committed any acts of self-harm in the past 12 months without suicidal intent
Cao et al.^36^	China	2104 (48.9%)	13.8 ± 1.7	stratified cluster sampling	Self-report	Self-report
					Have you been bullied or threatened at school in the past year? Such as being ignored, criticized, beaten up by classmates, etc."	Participants were asked,‘Have you deliberately harmed or injured yourself without intending to end your own life during the past 12 months?
Chen et al. 2019^37^	China	7129 (41.8%)	15.48 ± 1.65	Cluster sampling	Self-report	Self-report assessed using the Ottawa Self-injury Inventory(OSI)
					Five items from the revised Olweus Bully/Victim Questionnaire	
Zhang et al.^38^	China	1366 (42.6%)	19.67 ± 4.92	Convenience sampling	Self-report assessed using the Middle School Students Bullying Scale (MSSBS)	Self-report assessed using the Adolescent,Self-injury Scale (ASS)
Esposito et al.^39^	Italy	640 (60.5%)	15.60 ± 1.65	Convenience sampling	Self-report	Self-report
					Revised bully/victim questionnaire	NSSI was assessed through a six-item scale measuring how frequently during the last 6 months, adolescents intentionally engaged in several types of self-injurious behaviors without suicidal intentions (such as cutting, burning, or hitting oneself)
Gaspar et al.^40^	Portugal	3262 (54%)	14.8 ± 1.2	Random sampling from 36 national groups of public schools	Self-report	Self-report
					How many times have you taken part in bullying other student/ been bullied in the last 2 months?	During the past 12 months, how many times have you hurt yourself on purpose?
Yang et al.^41^	China	2380 (46.2%)	Junior school student	Multi-stage stratified random cluster sampling	Self-report	Self-report
					Revised bully/victim questionnaire	Have you deliberately harmed or injured yourself without intending to end your own life during the past 12 months?
Zhang et al.^42^	China	1497 (52.7%)	12.01–16.41	Stratified Cluster Random Sampling	Self-report	Self-report
					Middle School Students Bullying Scale (MSSBS)	Adolescent,Self-injury Scale (ASS)
Zhou et al.^43^	China	4434 (49.95%)	14.38 ± 1.68	Cluster sampling	Self-report	Self-report assessed using the Ottawa Self-injury Inventory (OSI)
					Revised bully/victim questionnaire	
Wang et al.^44^	China	878 (50.91%)	13.53 ± 1.08	Cluster random sampling	Self-report peer victimization was assessed with an adapted version of the Multidimensional Peer-Victimization Scale (MPVS)	Self-report NSSI was measured with the Deliberate Self-Harm Inventory (DSHI)
Tong et al.^45^	China	338 (50.9%)	Junior school student	Convenience sampling	Self-report	Self-report
					The revised Olweus Bully/Victim Questionnaire	During the past 12 months, how many times have you hurt yourself on purpose?
Mossige et al.^46^	Norway	6979 (58.4%)	18–19	Random sampling	Self-report Verbal bullying and threats of harm by peers and being injured by violence perpetrated by peers or by other young strangers	Self-report Participants report three self-harming behaviors. Reporting self-injury but not suicidal ideation or suicide attempt counts as NSSI
Lee et al.^47^	Korea	1674 (36.5%)	16.6 ± 0.5	Convenience sampling	Self-report	Self-report
					We asked students three items about their experiences with school violence using self-reported lifetime incidence of school bullying, reported as ‘Yes’ or ‘No’	NSSI was assessed by the Deliberate Self-Harm Inventory (DSHI)
Wu et al.^48^	China	813 (43%)	13.15	Convenience sampling	Self-report	Self-report
					The School Bullying/Victimization Scale was used to assess adolescents’ Bullying Perpetration/Victimization	“In the past 6 months, have you engaged in the following behaviors to deliberately harm yourself, but without suicidal intent?”

### Coding

Twenty-nine studies with 54 independent effect sizes were included in the meta-analysis. If the article included multiple independent samples (e.g., different countries, men and women), these samples were included separately in this study. The odds ratios (ORs) were coded as the effect size measure in the study. Three studies that did not report the OR of all samples but provided enough data for us to calculate the OR^29,32,46^. Several studies^13,14,23–25,27,30,31,37–39,42–45,48^ provided correlations instead of ORs. For these studies, Comprehensive Meta-analysis (Biostat; http://www.metaanalysis.com/index.php) was applied to convert correlations into ORs. If an article included both adjusted and unadjusted effect sizes, then including an effect size adjusted for most confounders would be our choice. Several articles offered different ORs for different levels of bullying behaviour (e.g., occasional, repetitive), or divided bullying behaviour into different forms (e.g., physical, verbal, or relational). For these studies, children or adolescents who had experienced any form of bullying behaviour into a group were combined and compared them with children or adolescents who did not engage in bullying. All studies provide data on the NSSI risk of victims, ten studies^12–14,32,34,39,40,42,47,48^ provide data on bullies, and six studies provide data on bully-victims ^12,32,34,39,47,48^. Two researchers coded the included studies, including study location, sample size, age, sampling method, data collection method, and effect sizes. In case of disagreement, a decision was discussed with a third researcher. Before the discussion, the agreement rate was 92.1%.

### Statistical analysis

Analyses were performed with the Comprehensive Meta-analysis. OR was used as the effect size of this meta-analysis. Other effect sizes, such as Pearson correlation coefficients, were converted to ORs prior to analysis. A Random effect model was used for data analysis. The random effect model is more suitable for the current meta-analysis than the fixed effect model because it does not assume the common potential effect size of all studies included in the meta-analysis. We performed meta-regression using the average age of the participants as a moderating factor. The average age of the subjects was not described in four studies^27,30,41,45^, but the age range of these four studies was relatively small, so we used the average of the age range instead of these. We compared the effects of studies involving only girls with those involving only boys to analyse the moderating role of gender on the association between bullying behaviour and NSSI. In addition, subgroup analysis was used to compare whether the effects of articles with random sampling designs were different from those with non-random sampling designs.

To test potential publication bias, Orwin's fail-safe N^49^, Duval and Tweedie’s trim-and-fill analysis^50^, and Egger’s regression intercept^51^ were calculated. The first estimates how many studies with insignificant results would be required to offset the effect size obtained in a meta-analysis. If many studies were needed, we might assume that although the effect size obtained through meta-analysis may be slightly overestimated, there is no obvious publication bias in its significant effect. The recommended rule is that the number of articles estimated by Orwin's fail-safe N procedure has to be more than 5K + 10,^52^, where K represents the number of effects included in the study. The Duvall and Tweedie trim and fill method estimates the effect size until the error distribution is very close to normal to provide a more unbiased estimate of the effect size than observed estimates. Egger's regression intercept used the linear regression method to evaluate the study effect size relative to its standard error to measure potential publication bias; Q-test and I^2^-test were used to detect the heterogeneity of the results^53^.

### Quality assessment

The quality assessment of the included studies was conducted using the JBI Checklist^54^. The options for each evaluation item were yes, no and unclear. A yes answer counted for one point, and the rest counted for zero. The higher the score, the higher the quality of the included literature. Two researchers evaluated the quality of the included literature and communicated with the third researcher if there was any disagreement. The evaluation results showed that the 29 articles included in this study were of medium to high quality (total score ≥ 6). For specific evaluation, see Supplementary file 3.

## Results

### Association between being bullied and NSSI

**Figure 2:**
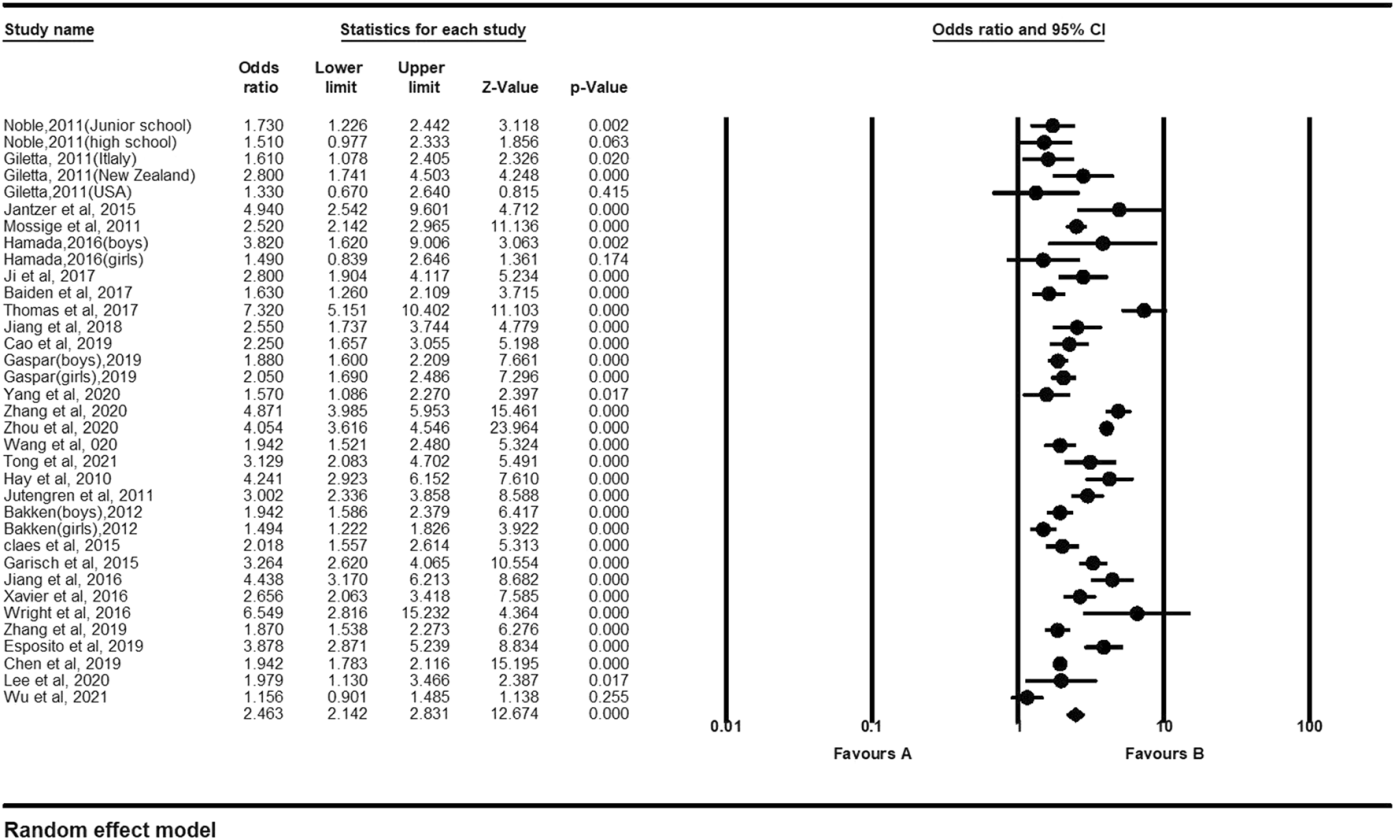
Forest plot for the effect size comparing the NSSI of victims with children not involved in bullying.

**Figure 3:**
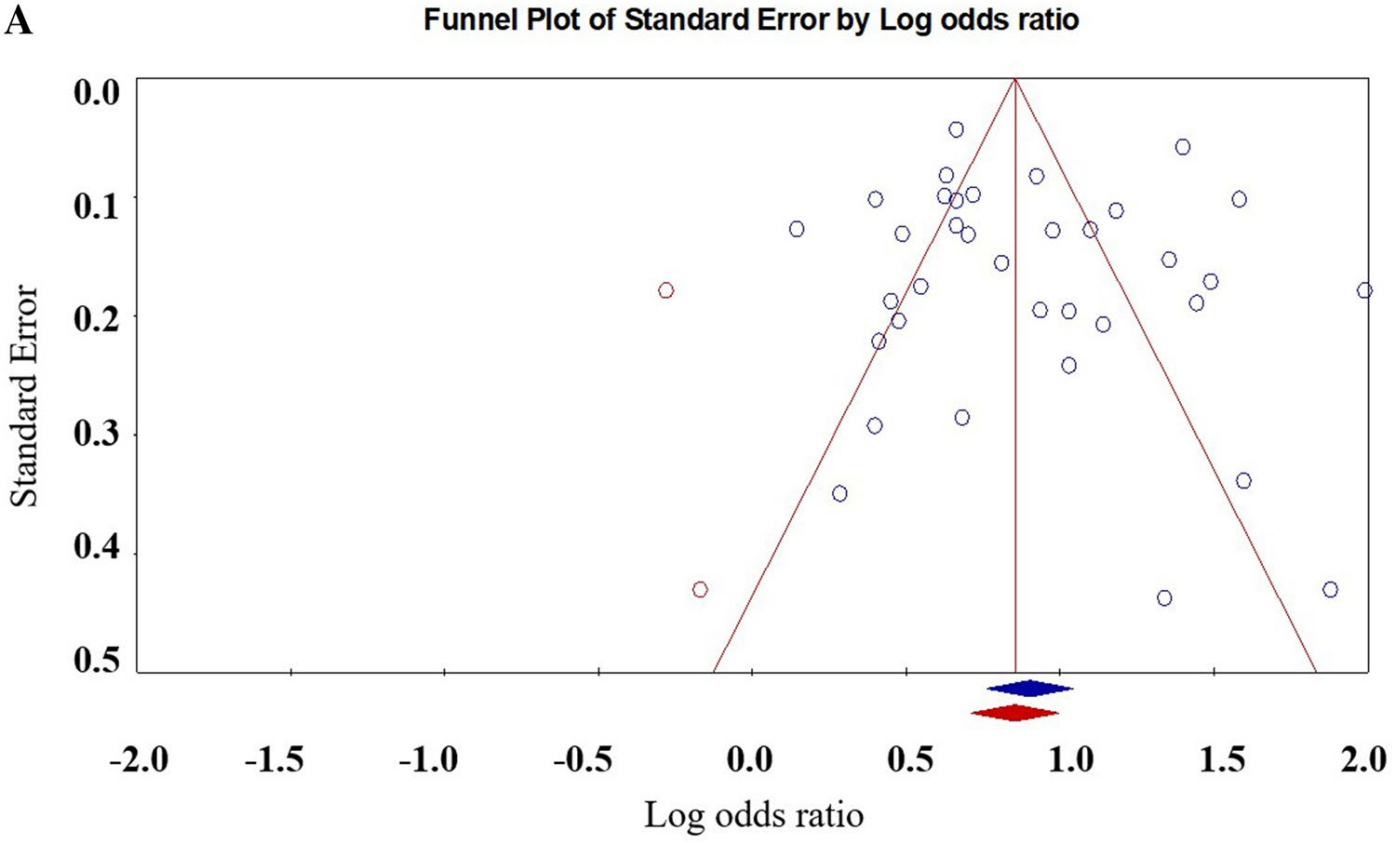
Filled funnel plot of the relationship between non-suicidal self-injury and being bullied.

Twenty-nine articles that met the inclusion criteria included 35 effect sizes with a total of 53,501 youths. The results showed that victims were significantly associated with NSSI [OR: 2.46 (95% CI 2.14–2.83); *p* < 0.001]. Table 2 shows the details. Figure 2 shows the forest plot. Egger regression analysis showed that t = 0.44, *p* = 0.66. The Duval and Tweedle trim-and-fill procedure indicated that two studies needed to be imputed, but the adjusted effect size (OR 2.34; 95% CI 2.03–2.70) was consistent with the size observed in existing studies (Fig. 3). Orwin Fail safe N shows that an additional 290 studies of invalid effect sizes are needed to reduce this combined effect size to an insignificant value (5k + 10 benchmark = 185). The Egger regression, the Duvall and Tweedle trim-and-fill procedure, and the Orwin fail-safe N indicated that the impact of publication bias was negligible for the meta-analyses. The methodological characteristics of the study were analysed through the moderating effect analysis. Studies using non-random sampling designs [OR = 2.73 (95% CI 2.28–3.26)] were not significantly different from those using random sampling designs [OR = 2.09 (95% CI 1.84–2.37)], [Q = 1.51, *p* = 0.22]. Studies using only boy samples [OR = 2.34 (95% CI 1.71–3.21)] were not significantly different from studies using only girl samples in effect sizes [OR = 1.71 (95% CI 1.33–2.20)], (Q = 2.31, *p* = 0.13). In addition, a meta-regression analysis of age shows that the older the age, the lower the risk of NSSI for the bullied (z = − 3.74, *p* = 0.00).

**Table 2:** Results of the Meta-analyses Between Bullying Roles and NSSI.

Role	No. of studies	No. of effect sizes	No. of adolescents	Odds ratio (95% CI)	Q	I^2^	Orwin’s fail-safe N
Victims	29	35	53,501	2.46 (2.14–2.83)	359.558	90.544	290
Bullies	10	12	14,253	2.12 (1.37–3.27)	152.03	92.76	89
Bully-Victims	6	7	8613	2.98 (1.85–4.82)	34.31	82.51	72

### Association between active bullying and NSSI

**Figure 4:**
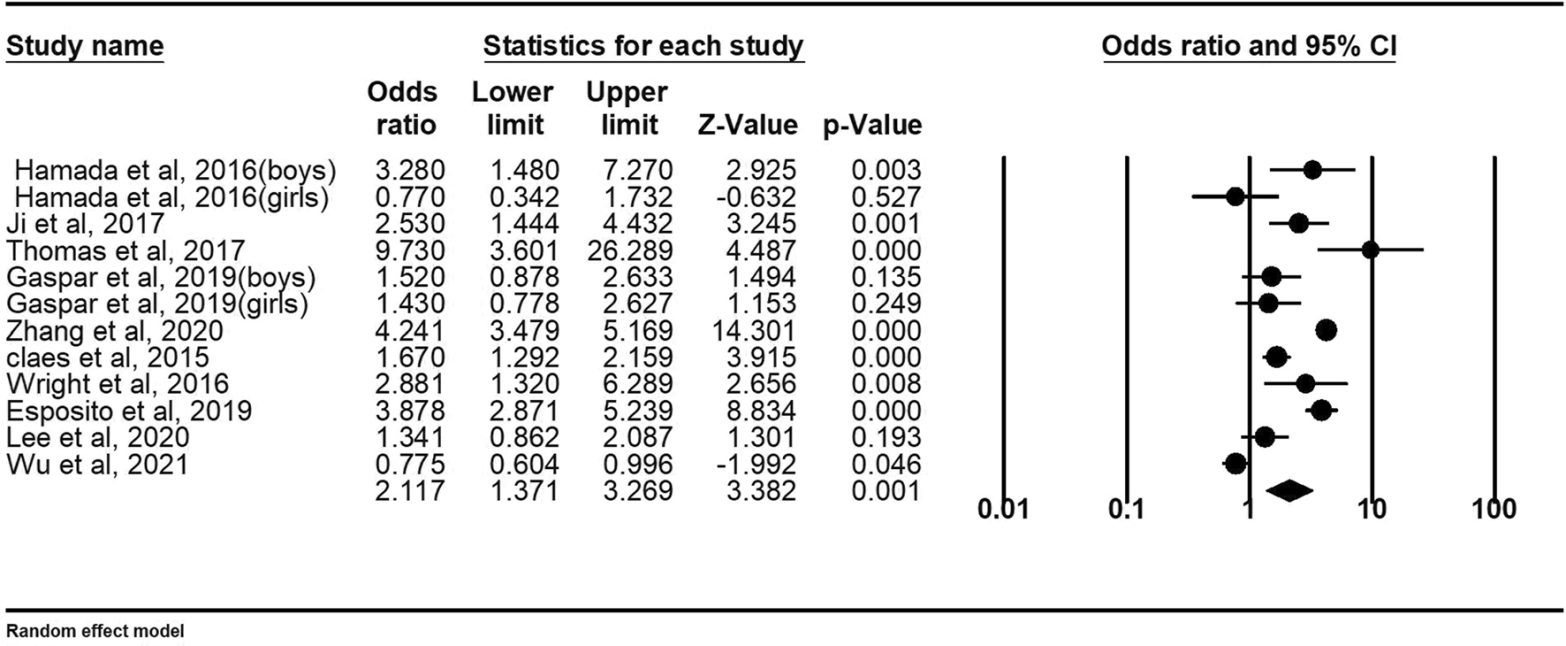
Forest plot for the effect size comparing the NSSI of bullies with children not involved in bullying.

**Figure 5:**
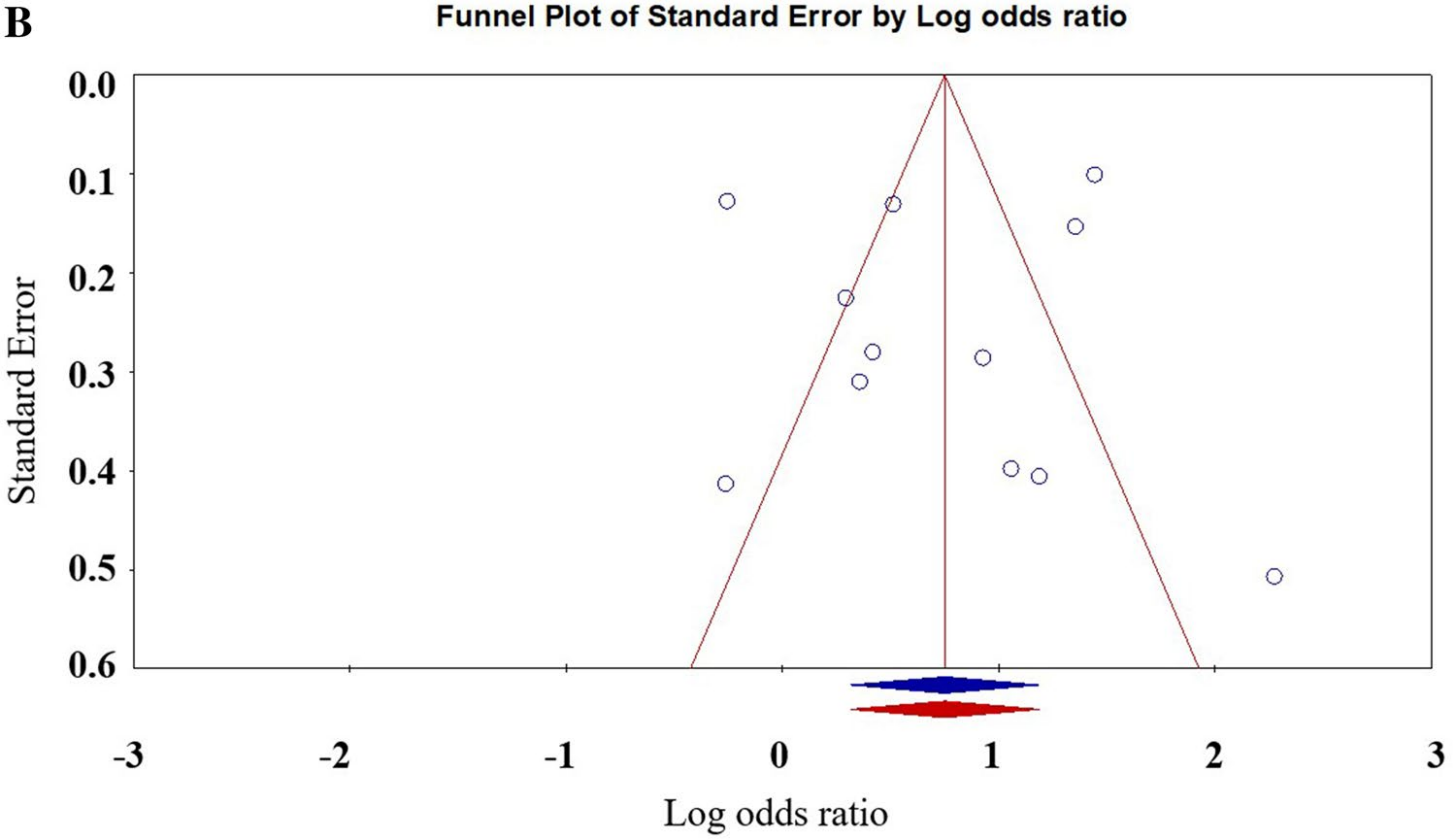
Filled funnel plot of the relationship between non-suicidal self-injury and active bullying.

Eight studies provided data on bullying youths. Meta-analyses indicate that bullying youths are more likely to develop NSSI than youth who are not involved in bullying [OR: 2.12 (95% CI 1.37–3.27); *p* < 0.001]. See Table 2 for details. Figure 4 shows the forest plot. Egger regression analysis shows that t = 0.25, *p* = 0.80. The Duval and Tweedle trim-and-fill procedure suggested that no additional research is needed (Fig. 5). Orwin Failsafe N showed that an additional 89 studies of invalid effect sizes are needed to reduce this combined effect size to an insignificant value (“5k + 10” benchmark = 70). The Egger regression, the Duvall and Tweedle trim-and-fill procedure, and the Orwin fail-safe N show no publication bias. Studies using non-random sampling designs [OR = 2.91 (95% CI 1.39–6.12)] were not significantly different from those using random sampling designs [OR = 1.79 (95% CI 1.12–2.86)], [Q = 1.19, *p* = 0.28]. The effect sizes of samples with only boys [OR = 2.11 (95% CI 1.00–4.45)] did not differ from the effect sizes of samples with only girls [OR = 1.11 (95% CI 0.62–2.02)], [Q = 1.72, *p* = 0.18]. The meta regression showed that the moderating effect of age is not statistically significant (z = 0.69, *p* = 0.49).

### Association between bully-victims and NSSI

**Figure 6:**
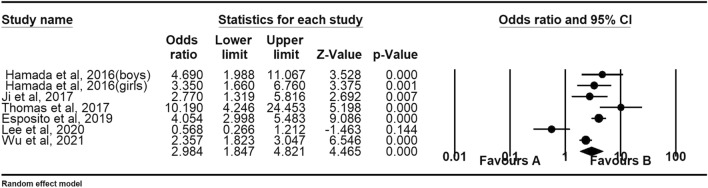
Forest plot for the effect sizes comparing the NSSI of Bully-Victims with children not involved in bullying.

**Figure 7:**
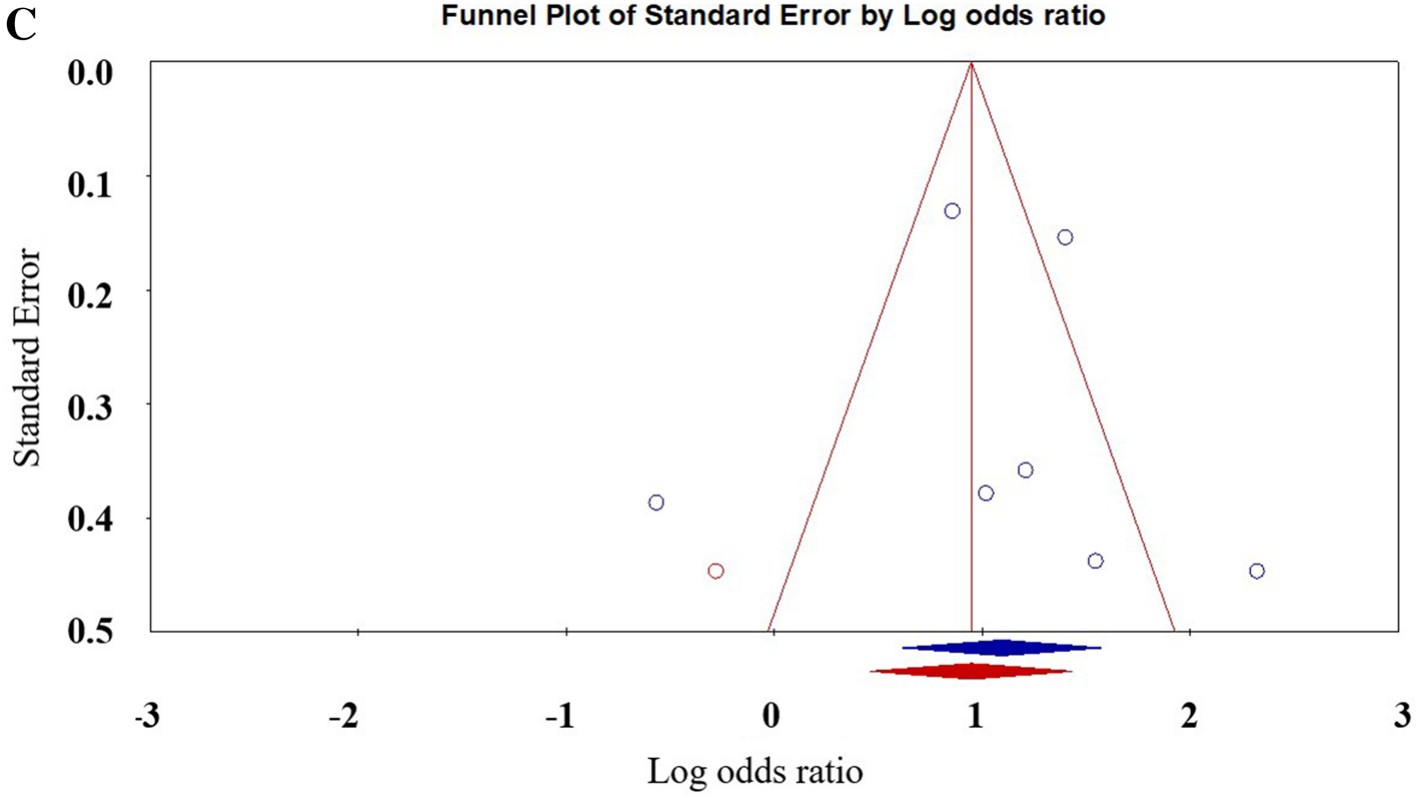
Filled funnel plot of the relationship between non-suicidal self-injury and bully-Victims.

Finally, six studies examining the risk of bullying-victims developing NSSI compared with non-participating peers were analysed Meta-analysis shows that bullying-victims are more likely to engage in NSSI behaviour than non-involved peers [OR: 2.98 (95% CI 1.85–4.82); *p* < 0.001]. Table 1 shows the details. Figure 6 shows the forest plot. There was no evidence of publication bias. Egger regression analysis shows that t = 0.13, *p* = 0.90. The Duval and Tweedle trim-and-fill procedure indicated that 1 study needed to be imputed, but the adjusted effect size (OR 2.57; 95% CI 1.59–4.19) was consistent with the size observed in existing studies (Fig. 7). An additional 72 studies with null effect sizes would be needed to attenuate this omnibus effect size to a nonsignificant value (“5k + 10” benchmark = 45). Among the studies that included the bullying-victim group, only one study^8^ had independent male and female data, and only one study^8^ used a random sampling design, so meta-analysis was temporarily impossible. Meta-regression shows that age is not a significant moderating variable (z = 0.84, *p* = 0.40).

## Discussion

Meta-analysis is an invaluable tool to integrate previous research, illuminate research gaps, and define priorities for future research. Twenty-nine articles were finally included in this study, and the quality evaluation results showed that they were of moderate to high quality. Meta-analysis results indicated that victims, bullies, and bully-victims were at higher risk of developing NSSI than their uninvolved peers. This is consistent with previous research results^55^. The reason may be that tensions can cause people to produce depression, anxiety, anger and other negative emotions. To release these negative emotions, dangerous behaviors are adopted such as self-injury and aggression to deal with them^56^. According to Agnew's general strain theory (GST), bullying incidents have physical and psychological effects on teenagers. It can cause lasting harm and the feeling of social injustice. In the face of such negative life events, deviant behaviours (such as violence and self-injury) become the most effective way to manage negative emotions^57^. Being bullied is an important source of stress^58^. Teenagers who have experienced this behaviour often have difficulty adapting to school or society, which may increase the risk of implementing dangerous behaviours (such as NSSI). This view has been confirmed by some studies^30^. The interpersonal model can also be used to explain the significant relationship between bullying and NSSI. This model considers NSSI as a negative coping strategy to reduce the pressure or tension caused by adverse interpersonal events^59^. For adolescents, bullying and being bullied are relatively common negative interpersonal events^60^. Bad interpersonal events may cause serious emotional distress. If adolescents cannot adjust in time, NSSI behaviours may occur, which will make the adolescent’s emotional and interpersonal communication tendencies desperate. Once NSSI promotes personal emotional comfort, it is reused this way to release emotions^61^.

The methodological characteristic that was studied as a moderator, namely, sampling strategy, was found not to significantly moderate the relationship between bullying and NSSI. This suggests that the effect size is not inflated by the inclusion of studies with relatively less rigorous methodologies. To analyse the moderating role of age in the relationship between bullying behavior and non-suicidal self-harm, a meta-regression was performed. The regression results found that in the bullied group, the older the age was, the lower the risk of NSSI. The results do not necessarily mean that younger children are more susceptible to bullying, however, peer victimization has been found to be more frequent in younger people than in older people^62^. In the current meta-analysis, it is possible that younger victims simply experienced more bullying incidents and thus experienced more NSSI than older victims. In addition, younger children may lack coping strategies to appropriately deal with peer victimization and are therefore more likely to develop problems as a result of peer victimization. Finally, no difference was found in effect size between boys and girls. One study found that being female was protective against bullying^63^, and another found that it was a risk factor for later bullying^64^. The literature for children indicates that bullies appear to most often be boys, but both males and females tend to be victims^65^. In their study of the relationship between bullying and suicidal ideation in adolescents, Brunstein Klomek et al.^66^ found that for girls, being bullied was associated with an immediate increased risk of suicidal ideation, while for boys, only chronic bullying was associated with suicidal ideation. However, similar gender differences were not verified in this meta-analysis, which may be related to the greater similarity in bullying behaviours experienced by boys and girls. In the bullying-victim group, gender differences in the relationship between bullying-victimization and NSSI could not be verified because the included articles did not provide the effect size needed to address this relationship. In conclusion, the current study suggests that NSSI in both boys and girls may be associated with bullying in equally strong ways.

## Limitations and future implications

Although there are some highlights, several limitations must be considered. First, although the included studies were of relatively high quality, most of the data were collected in the form of self-reports by the subjects, which may cause recall bias. One possible limitation of this method is that it requires the interviewee to have a good level of self-awareness. In addition, some children who are bullied may tend to deny their status, while active bullies may be reluctant to admit that they are real bullies. Finally, the correlation between data from the same source (that is, when both the bullying experience and NSSI are self-reported by the child) may be exaggerated by the variance of the commonly used methods. One possibility for future research is that information should be collected through multiple independent information providers, such as children themselves, their peers within the classroom, and their teachers or parents. Additionally, an assessment of adolescents’ physical health may also be useful. Second, our meta-analysis is based on a cross-sectional study, and can only conclude that bullying is related to NSSI and cannot make any claims about the causal relationship between bullying and NSSI. Therefore, more longitudinal studies are needed in the future to test whether involvement in bullying increases NSSI or whether NSSI increases bullying behaviour. Third, since this paper only considers peer-reviewed articles, some grey literature may be missed, which may affect the results to some extent. Therefore, future research may further consider the inclusion of high-quality grey literature.

Fourth, in this meta-analysis, very few studies have measured different forms of bullying (verbal bullying, physical bullying, etc.) or did not report separate analyses for different forms of bullying. Although these two forms of bullying are not independent experiences types in the lives of children and adolescents. More precisely, they are two partially overlapping forms of harassment. However, recent research shows the importance of distinguishing between these two forms of bullying because they may have different relationships with personal adaptation^67,68^. Future research should further analyse the negative effects of physical and relationship or indirect bullying experiences on NSSI. The current analysis excludes research that only focuses on cyber bullying to focus on the risks of traditional forms of bullying; however, people know little about cyber bullying and cyber victimization, but they have become increasingly important in modern culture^69^. Future research should aim to determine whether the risk of cyberbullying and cyber victimization from NSSI is different from the traditional forms of these behaviours. Finally, only six studies were included in the bully-victims analysis. It is best to update the results on bully-victims when more research is available. Due to the small number of studies included and lack of information, we were unable to analyse the moderating effect of sampling strategy gender among bullying—victims.

## Conclusions

Adolescent NSSI is a significant public health concern at the international level. Bullying behaviour is also an increasing concern particularly due to its associations with adverse health and behavioural outcomes. The negative outcomes of bullying behaviour, coupled with the negative outcomes of NSSI make both issues incredibly deleterious and pervasive. This study examined associations between bullying behaviour and NSSI. The findings support the fact that NSSI behaviours are related to bullying behaviors, and teenagers who participate in bullying may report more NSSI behaviours than those who do not. Considering the significant relationship between bullying and adolescent NSSI, NSSI problems in children and adolescents can be minimized by reducing bullying among children.

## Clinical implications

The research reviewed supports the fact that bullies, victims, and bullying-victims are all associated with the risk of developing NSSI in adolescents. This result has significant implications for paediatricians, psychologists, and other health care professionals. It is very important that these professionals be able to identify adolescents who are at risk of being involved in NSSI because the potential negative health, psychological, and educational consequences are far-reaching. Based on significant association between bullying and psychosomatic problems, it has been suggested that when encountering psychosomatic complaints in children or adolescents, practitioners should regularly ask children or their parents about bullying to assess emotional function and peer experience^70^. Based on the results of the current study, we would like to extend this advice to NSSI. Therapy can also be provided for youth involved in bullying. Research has shown that when people receive the intervention of Treatment for Self-Injurious Behaviors (T-SIB) intervention, the frequency of NSSI decreases significantly^71^. At the same time, teenagers may choose not to disclose their involvement in bullying given the significant relation. NSSI could be used as a warning sign of children's involvement in bullying. Finally, interventions to reduce bullying should be considered. The need to support adolescents reporting bullying is a key priority in both school- and community-based settings in order to prevent further perpetration and/or victimization. Furthermore, understanding the factors that influence bullying perpetration and victimization is critical in reducing their incidence and persistence. Applying prevention science approaches that integrate population intervention studies and developmental research to examine modifiable risk and protective factors will assist in identifying and understanding these factors. Such understandings can then be used to inform evidence-based prevention and early intervention approaches targeted at bullying and its adverse consequences for both perpetrators and victims.

## Supplementary Information

Supplementary Information 1.

Supplementary Information 2.

Supplementary Information 3.

## Data Availability

The datasets used and/or analysed during the current study available from the corresponding author on reasonable request.
