# Supplementary material for: A meta-analysis of the relationship between bullying and non-suicidal self-injury among children and adolescents
Source: Sci Rep. 2022 Oct 14;12:17285. doi: 10.1038/s41598-022-22122-2 (PMC9568539; doi:10.1038/s41598-022-22122-2)
Supplement: Supplementary file 2 — Supplementary Information 2. [file 41598_2022_22122_MOESM2_ESM.docx]

**SEARCH STRATEGY IN PUBMED DATABASE**

| **Search** | **Query** | **Results** | **Time** |
| --- | --- | --- | --- |
| **#1** | **Search: (((((((bully*[Title/Abstract]) OR (bullie*[Title/Abstract])) OR (peer victim*[Title/Abstract])) OR (peer harassment[Title/Abstract])) OR (peer aggression[Title/Abstract])) OR (school violence[Title/Abstract])) OR (mobbing[Title/Abstract])) OR (ragging[Title/Abstract])** | **8,976** | **06:25:24** |
| **#2** | **Search: ((((((((((parasuicid*[Title/Abstract]) OR (self-harm*[Title/Abstract])) OR (self-injur*[Title/Abstract])) OR (self-cut*[Title/Abstract])) OR (self-destruct*[Title/Abstract])) OR (auto-mutilat*[Title/Abstract])) OR (auto-destruct*[Title/Abstract])) OR (non-suicidal[Title/Abstract])) OR (self-mutilation[Title/Abstract])) OR (non-suicidal self-injury[Title/Abstract])) OR (NSSI[Title/Abstract])** | **16,367** | **06:27:36** |
| **#3** | **Search: ((((((((bully*[Title/Abstract]) OR (bullie*[Title/Abstract])) OR (peer victim*[Title/Abstract])) OR (peer harassment[Title/Abstract])) OR (peer aggression[Title/Abstract])) OR (school violence[Title/Abstract])) OR (mobbing[Title/Abstract])) OR (ragging[Title/Abstract])) AND (((((((((((parasuicid*[Title/Abstract]) OR (self-harm*[Title/Abstract])) OR (self-injur*[Title/Abstract])) OR (self-cut*[Title/Abstract])) OR (self-destruct*[Title/Abstract])) OR (auto-mutilat*[Title/Abstract])) OR (auto-destruct*[Title/Abstract])) OR (non-suicidal[Title/Abstract])) OR (self-mutilation[Title/Abstract])) OR (non-suicidal self-injury[Title/Abstract])) OR (NSSI[Title/Abstract]))** | **231** | **06:27:57** |

**SEARCH STRATEGY IN EMBASE DATABASE**

| **History** | **Query** | **Results** |
| --- | --- | --- |
| **#1** | **'bully*':ab,ti** | **8,011** |
| **#2** | **'bullie*':ab,ti** | **2,342** |
| **#3** | **'peer victim*':ab,ti** | **1,189** |
| **#4** | **'peer harassment':ab,ti** | **52** |
| **#5** | **'peer aggression':ab,ti** | **162** |
| **#6** | **'school violence':ab,ti** | **390** |
| **#7** | **'mobbing':ab,ti** | **463** |
| **#8** | **'ragging':ab,ti** | **28** |
| **#9** | **#1 OR #2 OR #3 OR #4 OR #5 OR #6 OR #7 OR #8** | **10,359** |
| **#10** | **'parasuicid*':ab,ti** | **851** |
| **#11** | **'self-harm*':ab,ti** | **8,940** |
| **#12** | **'self-injur*':ab,ti** | **6,675** |
| **#13** | **'self-cut*':ab,ti** | **295** |
| **#14** | **'self-destruct*'** | **2,522** |
| **#15** | **'auto-mutilat*':ab,ti** | **28** |
| **#16** | **'auto-destruct*':ab,ti** | **69** |
| **#17** | **'non-suicidal':ab,ti** | **1,791** |
| **#18** | **'self-mutilation':ab,ti** | **1,771** |
| **#19** | **'non-suicidal self-injury':ab,ti** | **1,028** |
| **#20** | **'nssi':ab,ti** | **1,534** |
| **#21** | **#10 OR #11 OR #12 OR #13 OR #14 OR #15 OR #16 OR #17 OR #18 OR #19 OR #20** | **20,220** |
| **#21** | **#9 AND #21** | **280** |
