# Supplementary material for: A meta-analysis of the relationship between bullying and non-suicidal self-injury among children and adolescents
Source: Sci Rep. 2022 Oct 14;12:17285. doi: 10.1038/s41598-022-22122-2 (PMC9568539; doi:10.1038/s41598-022-22122-2)
Supplement: Supplementary file 3 — Supplementary Information 3. [file 41598_2022_22122_MOESM3_ESM.docx]

| Study | ① | ② | ③ | ④ | ⑤ | ⑥ | ⑦ | ⑧ | ⑨ | ⑩ | ⑪ |
| --- | --- | --- | --- | --- | --- | --- | --- | --- | --- | --- | --- |
| Hay et al.2010 | Y | Y | Y | Y | U | N | N | N | N | Y | N |
| Noble et al 2011 | Y | Y | Y | Y | U | N | N | N | N | Y | N |
| Bakken et al 2012 | Y | Y | Y | Y | U | N | Y | N | N | Y | N |
| Giletta et al 2012 | Y | Y | Y | Y | U | N | N | N | N | Y | N |
| Claes et al 2015 | Y | Y | Y | Y | U | Y | Y | Y | N | Y | N |
| Jantzer et al 2015 | Y | Y | Y | Y | U | Y | N | N | N | Y | N |
| Garisch et al 2016 | Y | Y | Y | Y | U | Y | N | N | N | Y | N |
| Hamada et al 2016 | Y | Y | Y | Y | U | N | N | N | N | Y | N |
| Jiang et al 2016 | Y | Y | Y | Y | U | N | N | N | N | Y | N |
| Xavier et al 2016 | Y | Y | Y | Y | U | Y | Y | Y | N | Y | N |
| Wright 2016 | Y | Y | Y | Y | U | Y | N | Y | N | N | N |
| Ji et al 2017 | Y | Y | Y | Y | U | Y | Y | Y | N | Y | N |
| Baiden et al 2017 | Y | Y | Y | Y | U | Y | Y | Y | N | Y | N |
| Thomas et al 2017 | Y | Y | Y | Y | U | N | N | N | N | Y | N |
| Jiang et al 2018 | Y | Y | Y | Y | U | N | Y | N | N | Y | N |
| Cao et al 2019 | Y | Y | Y | Y | U | Y | N | Y | N | Y | N |
| Chen et al 2019 | Y | Y | Y | Y | U | Y | N | N | N | Y | N |
| Zhang et al 2019 | Y | Y | Y | Y | U | Y | N | N | N | Y | N |
| Esposito et al 2019 | Y | Y | Y | Y | U | N | N | N | N | Y | N |
| Gaspar et al 2019 | Y | Y | Y | Y | U | N | N | N | N | Y | N |
| Yang et al 2020 | Y | Y | Y | Y | U | Y | Y | N | N | Y | N |
| Zhang et al 2020 | Y | Y | Y | Y | U | Y | Y | Y | N | N | N |
| Zhou et al 2020 | Y | Y | Y | Y | U | Y | N | Y | N | Y | N |
| Wang et al 2020 | Y | Y | Y | Y | U | Y | Y | Y | N | Y | N |
| Tong et al 2021 | Y | Y | Y | Y | U | N | N | N | N | Y | N |
| Mossige et al 2016 | Y | Y | Y | Y | U | N | Y | N | N | Y | N |
| Noble et al 2011 | Y | Y | Y | Y | U | Y | Y | Y | N | Y | N |
| Bakken et al 2012 | Y | Y | Y | Y | U | N | Y | N | N | Y | N |
| Lee et al, 2020 | Y | Y | Y | Y | U | Y | Y | Y | N | Y | N |
| Wu et al, 2021 | Y | Y | Y | Y | U | Y | Y | Y | N | N | N |

Y:Yes;N:No;U:Uclear; ①Define the source of information (survey, record review)；②List inclusion and exclusion criteria for exposed and unexposed subjects(cases and controls) or refer to previous publications; ③Indicate time period used for identifying patients; ④indicate whether or not subjects were consecutive if not population-based; ⑤indicate if evaluators of subjective components of study were masked to other aspects of the status of the participants; ⑥Describe any assessments undertaken for quality assurance purposes(e.g., test/retest of primary outcome measurements); ⑦Explain any patient exclusions from analysis; ⑧Describe how confounding was assessed and/or controlled; ⑨If applicable, explain how missing data were handled in the analysis; ⑩Summarize patient response rates and completeness of data collection; ⑪Clarify what follow-up, was expected and percentage of patients for which incomplete data or follow-up was obtained.
